# Distinct correlations between lipogenic gene expression and fatty acid composition of subcutaneous fat among cattle breeds

**DOI:** 10.1186/s12917-018-1481-5

**Published:** 2018-05-23

**Authors:** David Gamarra, Noelia Aldai, Aisaku Arakawa, Luis Javier R. Barron, Andrés López-Oceja, Marian M. de Pancorbo, Masaaki Taniguchi

**Affiliations:** 1 Biomics Research Group, University of the Basque Country (UPV/EHU), 01006 Vitoria-Gasteiz, Spain; 2 Lactiker Research Group; Lascaray Research Center, University of the Basque Country (UPV/EHU), 01006 Vitoria-Gasteiz, Spain; 3 Animal Genome Unit, Institute of Livestock and Grassland Science, National Agriculture and Food Research Organization (NARO), Tsukuba, 305-0901 Japan

**Keywords:** Cattle, Desaturation index, Fatty acid composition, Gene expression, Lipid metabolism, *SCD*, Subcutaneous adipose tissue, *SREBP*, ∆9-desaturase

## Abstract

**Background:**

The fatty acid (FA) composition of adipose tissue influences the nutritional quality of meat products. The unsaturation level of FAs is determined by fatty acid desaturases such as stearoyl-CoA desaturases (SCDs), which are under control of the transcription factor sterol regulatory element-binding protein (SREBP). Differences in *SCD* genotype may thus confer variations in lipid metabolism and FA content among cattle breeds. This study investigated correlations between FA composition and lipogenic gene expression levels in the subcutaneous adipose tissue of beef cattle breeds of different gender from the Basque region of northern Spain. Pirenaica is the most important beef cattle breed in northern Spain, while Salers cattle and Holstein-Friesian cull cows are also an integral part of the regional beef supply.

**Results:**

Pirenaica heifers showed higher monounsaturated FA (MUFA) and conjugated linoleic acid (CLA) contents in subcutaneous adipose tissue than other breeds (*P* < 0.001). Alternatively, Salers bulls produced the highest oleic acid content, followed by Pirenaica heifers (*P* < 0.001). There was substantial variability in *SCD* gene expression among breeds, consistent with these differences in MUFA and CLA content. Correlations between *SCD1* expression and most FA desaturation indexes (DIs) were positive in Salers (*P* < 0.05) and Pirenaica bulls, while, in general, *SCD5* expression showed few significant correlations with DIs. There was a significant linear correlation between *SCD1* and *SRBEP1* in all breeds, suggesting strong regulation of *SCD1* expression by *SRBEP1*. Pirenaica heifers showed a stronger correlation between *SCD1* and *SREBP1* than Pirenaica bulls. We also observed a opposite relationship between *SCD1* and *SCD5* expression levels and opposite associations of isoform expression levels with the ∆9 desaturation indexes.

**Conclusions:**

These results suggest that the relationships between FA composition and lipogenic gene expression are influenced by breed and sex. The opposite relationship between *SCD* isoforms suggests a compensatory regulation of total SCD activity, while opposite relationships between *SCD* isoforms and desaturation indexes, specially 9*c*-14:1 DI, previously reported as an indicator of SCD activity, may reflect distinct activities of SCD1 and SCD5 in regulation of FA content. These findings may be useful for beef/dairy breeding and feeding programs to supply nutritionally favorable products.

## Background

In recent years, consumers have expressed growing concern regarding the amount and types of dietary fat due to reported deleterious health effects of saturated and *trans* fatty acids (FAs) [1]. On the other hand, monounsaturated FAs (MUFAs) and polyunsaturated FAs (PUFAs) are recognized as beneficial for human health [2]. The FA composition of meat influences the lipid melting point [3], and an increase in the ratio of MUFA to saturated FA (SFA) increases fat softness, thereby improving palatability. Thus, enhancing MUFA content improves both the quality and nutritional value of animal products [4].

Pirenaica is the most important beef cattle breed raised in the Basque region of northern Spain and is highly appreciated both for its value as a genetic resource as well as for the production system that has developed around it. In addition to Pirenaica, Salers, a rustic cattle breed used for beef production, has grown in importance due to its ready adaptability to local management and environmental conditions [5]. Finally, Holstein-Friesian, primarily as cull dairy cows, are also an integral part of the regional beef supply chain.

There have been a number of studies investigating associations between lipogenic enzyme genotype and FA composition in cattle. In the subcutaneous and intramuscular fat depots of beef cattle, the majority of SFA conversion to MUFA is catalyzed by stearoyl-CoA desaturase (SCD, EC 1.14.19.1 or ∆9-desaturase) [6]. In addition, SCD enzyme can also catalyze the conversion of substrates like vaccenic acid (11 *t*-18:1) to its corresponding conjugated linoleic acid (CLA) isomer (9*c,*11 *t*-18:2 or rumenic acid). The association of *SCD*1 genotype with FA composition has been previously investigated in Japanese Black [7], Canadian Holstein [8], Fleckvieh [9] and crossbred cattle [10]. In addition to regulation of FA profile by *SCD1*, the novel ∆9-desaturase isoform *SCD5*, previously found in humans, has also been identified in cattle which shares 65% identity at the amino acid level [11]. Further, a relationship between genetic polymorphisms in *SCD5* and the ratio of SFA to unsaturated FA (UFA) has been reported in Holstein milk fat [12]. It thus appears that both bovine isoforms *SCD*1 and *SCD*5 contribute to FA composition. Therefore, the mechanism by which *SCD* isoforms are activated is a major determinant of FA composition and of great interest to breeders.

Da Costa et al. [13] reported a correlation between *SCD1* expression and FA composition of subcutaneous fat in Portuguese cattle, whereas the expression of *SCD5* and its relation to the subcutaneous FA profile was not investigated. The *SCD1* gene is controlled by the key transcription factor *SREBP1* [14]. In Japanese Black beef cattle, *SREBP1* polymorphisms have been associated with FA composition [15]. Alternatively, transcriptional regulation of bovine *SCD*5 remains unclear, although a recent study using human choriocarcinoma trophoblastic cells (JEG3) reported that *SREBP1* can bind to the *SCD5* promoter [16].

The complex associations between the biochemical pathways regulating fat content and genetic variability of lipogenic genes are not yet fully understood in European cattle breeds, although recent studies have begun to elucidate these relationships in a specific genetic background of Japanese Black cattle [17]. The objectives of the present study were to investigate the expression levels of three key genes controlling ∆9-desaturated FA content, *SCD1*, *SCD5*, and *SREBP1*, and their associations with the FA composition of subcutaneous adipose tissue from the major commercial cattle breeds produced in northern Spain, Pirenaica, Salers, and Holstein-Friesian. Based on the findings of this study, we discuss how these associations may give information on the mechanisms of the differences in meat quality among these cattle commercial types.

## Methods

### Sample collection

In the present study, cattle commercial types typically destined for meat production in northern Spain (Basque region) were examined. Sample collection was designed according to data from the Bovine Identification Document and inferred relationships (parentage and sibships) computed from 29 microsatellites (Software Colony 2.0.6.2) [18]. Neither parentage nor maternal half-sibs were observed, and paternal half-sibs were maintained at low frequencies (Pirenaica, 0.009; Salers 0.013; Holstein-Friesian, 0.019). A total of 100 subcutaneous adipose tissue samples were collected from pure breed cattle (13 Salers bulls, 37 Pirenaica bulls, 29 Pirenaica heifers, and 21 Holstein-Friesian) slaughtered in a local commercial abattoir (Urkaiko S. Coop., Zestoa, Gipuzkoa, Spain) during 12 days over 5 weeks in June and July 2014. Animals came from different farms [19].

Backfat samples were obtained from the left half carcass between the 5-6th ribs and stored in plastic bags with the air removed for FA analysis or preserved in RNAlater™ (Ambion, Austin, TX) for RNA analysis. All samples were transported to the laboratory in insulated coolers and stored at − 80 °C until analysis.

Salers and Pirenaica were yearling calves with similar age (average of 12.9 ± 1.4 months), while Holstein-Friesian were cull cows (70.0 ± 19.43 months) at slaughter, which are regular ages of commercial types used for beef production in the region. Hot carcasses of Salers and Pirenaica commercial bulls were of similar weight (average of 325 ± 38.4 kg) while carcasses of Pirenaica heifers and Holstein-Friesian cows were markedly lighter (291 ± 59.6 and 253 ± 33.1 kg, respectively).

In the abattoir, conformation and degree of fat cover of each carcass were recorded. European regulations were followed for carcass classification at 24 h post-mortem [20] including the EUROP scale for conformation and a 1-to-5 scale for fat cover scoring. Each level of both scales was divided in 3 sub-levels and transformed to a numerical scale ranging from 1 to 15, with 15 being the best conformation and the thickest fat cover.

### Fatty acid composition

A 50 mg sample of subcutaneous fat tissue was weighed, freeze-dried, and directly methylated with sodium methoxide (0.5 N methanolic base, Supelco Inc., Bellefonte, PA, USA) [21]. For quantitation, 1 mL of internal standard (23:0 methyl ester) was added prior to methylation, and FA methyl esters were analyzed by gas chromatography with flame ionization detection (GC/FID) using two complementary 100 m columns (SP-2560 [22] and SLB-IL111 [23]) and following the conditions and details reported in [24]. Main FA groups and potential Δ9 substrates, products, and inhibitors (10 *t*,12*c*-18:2; [25]) have been determined for the individual FAs measured in this study. From the potential substrates (14:0, 15:0, 16:0, 17:0, 18:0, 19:0, 20:0, 6-8 *t*-18:1, 11 *t*-18:1, 12 *t*-18:1, 13 *t*/14 *t*-18:1 and 15*c*-18:1) and products (9*c*-14:1, 9*c*-15:1, 9*c*-16:1, 9*c*-17:1, 9*c*-18:1, 9*c*-19:1, 9*c*-20:1, 7 *t*,9*c*-18:2, 9*c*,11 *t*-18:2, 9*c*,12 *t*-18:2, 9*c*,13 *t*-18:2, 9*c*,15*c*-18:2), individual desaturation indexes were calculated by the following formula:

$$ \mathrm{Desaturation}\ \mathrm{index}\ \left(\mathrm{DI}\right)=\left[\mathrm{product}\right]/\left(\left[\mathrm{substrate}\right]+\left[\mathrm{product}\right]\right). $$

Total DI (sum of all individual DIs) was also computed for each commercial type, while minor products and substrates (i.e., 11 *t*,15*c*-18:2 & 9*c*,11 *t*,15*c*-18:3 [26]) or below quantification limits were not considered in the present study.

### RNA extraction and quantitative real-time PCR

A 100 mg sample of frozen subcutaneous fat tissue was disrupted and simultaneously homogenized to fine powder with a mortar and pestle under liquid N_2_. Total RNA was extracted using the RNeasy Lipid Tissue kit (Qiagen Inc., Valencia, CA, USA) following the manufacturer’s instructions. An additional DNase digestion step was performed to remove any contaminating genomic DNA. Concentration and quality of the extracted RNA were assayed by measuring the 260 nm and 280 nm absorbance using a NanoDrop ND-1000 Spectrophotometer (Peqlab, Erlangen, Germany). Absorbance ratios (260/280) of all preparations were at least 1.8. Integrity of RNA was checked by denaturing agarose gel electrophoresis. Aliquots of RNA were stored at − 80 °C and dehydrated in RNAstable 96-Well Plates (Biomatrica, San Diego, CA, USA) for long-term storage. Reverse transcription was performed in a 30 μL final reaction volume containing 250 ng total RNA, 3.3 μL RNase/DNase-free water, 5 μL of 5 × RT buffer, 1.5 μL dNTPs, 0.8 μL RNAase inhibitor, 0.8 μL random primer, and 0.8 μL high efficient ReverTra Ace reverse transcriptase (TOYOBO, Osaka, Japan). Cycle parameters were 30 °C for 10 min, 42 °C for 20 min, 99 °C for 5 min, and 4 °C for 5 min. Custom TaqMan Assays (Applied Biosystems, Foster City, CA, USA) were conducted to measure the relative expression levels of bovine *SCD1*, *SCD5* and *SREBP1* using the primers and FAM/TAMRA probes reported in Table 1. Each candidate gene was amplified in multiplex with an internal control (18S rRNA Endogenous Control VIC/TAMRA Probe, Primer Limited) by the co-application reverse transcription method (Co-RT) [27]. This multiplexing approach guarantees the same conditions (thus equal amplification efficiency) and same reverse transcriptase activity for both genes, thereby yielding better normalization and reproducibility. The reaction mixture included primers (10 μM each), FAM-labeled probe (10 μM), 0.6 μL of 18S RNA Endogenous Standard containing VIC-labeled probe and limited primers, and 2 × TaqMan Gene Expression Master Mix (7.5 μL) (Applied Biosystems). Real-time PCR was performed in triplicate using the ABI Prism 7500 Sequence Detection System (Applied Biosystems, Foster City, CA, USA) with a standard two-step cycling program of 40 cycles at 95 °C for 15 s and 60 °C for 1 min. The average of the gene expression levels was used for further analyses. PCR efficiency was monitored by the increase in absolute fluorescence [28], mainly because this allows PCR efficiency calculation for individual samples/reactions and prevents problems arising from the use of standard curves. Raw data were obtained from the ABI Prism 7500 SDS software v1.4, exported in Rn format, and imported to LinRegPCR (Heart Failure Research Center, Amsterdam, the Netherlands). LinRegPCR determines baseline fluorescence, sets a window of linearity for each amplicon, and calculates the PCR efficiency (E) per sample and amplicons using an iterative algorithm. In this study, efficiencies were over 90% for all samples and correlation coefficients were higher than 0.99.

**Table 1:** Primer sequences, product sizes, and annealing temperatures of bovine genes analyzed by RT-PCR

Gene symbol [GenBank accession]	Primer sequence (5′ - 3′)	Product (bp)	Annealing temperature
*SCD*1	P: CCTCTGGAACATCACCAGCTTCTCGGC	106	60
[NM_173959]	F: GCTGTCAAAGAAAAGGGTTCCAC		
	R: AGCACAACAACAGGACACCAG		
*SCD*5	P: CAGAACCCGCTCGTCACCCTGGG	82	60
[NM_001076945]	F: CCCTATGACAAGCACATCAGCC		
	R: GATGGTAGTTATGGAAACCTTCACC		
*SREBP*1	P: CAGCCCCAGTCCTGGATCAGCCGA	83	60
[NM_001113302]	F: CTTGGAGCGAGCACTGAATTG		
	R: GGGCATCTGAGAACTCCTTGTC		

*P* = probe, *F* forward, *R* reverse

The comparative threshold cycle method (ΔCt) was employed to calculate relative gene expression based on the following formula:

$$ \Delta \mathrm{Ct}=\left({\mathrm{Ct}}_{\mathrm{target}\ \mathrm{gene}}\hbox{-} {\mathrm{Ct}}_{18\mathrm{S}\ \mathrm{rRNA}\ \mathrm{gene}}\right). $$

### Statistical analysis

Statistical analysis was conducted using IBM SPSS Statistics 22 for Windows (SPSS Inc., IBM Corporation, NY, USA). First of all, data was checked for normality and homoscedasticity. Then, the following general linear model *Y*_*ijk*_ = *μ* + *CT*_*i*_ + *A*_*j*_ + *HCW*_*k*_ + *e*_*ijk*_ was used for analysis of variance (ANOVA), including commercial type (CT; Salers bulls, Pirenaica bulls, Pirenaica heifers, Holstein-Friesian cows) as fixed effect and age at slaughter (A) and hot carcass weight (HCW) as covariates. The effect of sire was also checked but not included in the model as it was statistically not significant. LSD post hoc test was applied for multiple comparison of means among commercial breeds studied.

Simple linear regression analyses were also performed to investigate the relationship between genes (gene-gene) for each commercial type studied.

Finally, partial Pearson correlations coefficients adjusted for A and HCW were computed to determine the associations among gene expression (∆Ct) and FA (∆9 DIs) data.

Three significant figures were used to express the data, and significance was declared at *P* < 0.05.

## Results

### Carcass traits and fatty acid composition

Pirenaica heifer carcasses showed the highest fat cover, while those from Pirenaica bulls and Holstein-Friesian cows were lower, and carcasses from Salers showed an intermediate degree of fat cover (*P* < 0.001). In terms of FA composition, several significant differences in specific SFA species were found among commercial types (i.e., 14:0, 16:0, 19:0, 20:0 SFAs), but there were no significant differences in total SFA content. Pirenaica heifers exhibited the highest content of *cis*- and *trans*-MUFAs, and Salers bulls had higher *cis*-MUFA content than Pirenaica bulls (*P* < 0.001; Table 2). Accordingly, Pirenaica heifers showed the highest contents of 9*c*-14:1 and 9*c*-16:1, while 9*c*-17:1 and 9*c*-18:1 were the highest in Pirenaica heifers but also in Salers bulls. Additionally, Pirenaica heifers exhibited the highest content of individual *trans*-18:1 isomers, while vaccenic acid (11 *t*-18:1) and *trans*-12-octadecenoic acid (12 *t*-18:1) contents did not differ among commercial types. The total CLA content was highest in Pirenaica heifers (*P* < 0.01). However, no significant differences were observed in rumenic acid (9*c*,11 *t*-18:2), the major CLA isomer. The second major CLA isomer (7 *t*,9*c*-18:2), other non-conjugated dienes (6-8 *t*-18:1and 13 *t*/14 *t*-18:1), and potential products of Δ9-desaturation (9*c*,12 *t*-18:2, 9*c*,13 *t*-18:2 and 9*c*,15*c*-18:2) were significantly higher in Pirenaica heifers than in the other commercial types. In contrast, n-6 PUFA content was similar in Pirenaica heifers and Pirenaica bulls, and significantly lower in both compared to Salers bulls. Finally, the content of 10 *t*,12*c*-CLA, reported as an inhibitor of Δ9-desaturase, was higher in fat tissues of Pirenaica heifers than in other commercial types (*P* < 0.001; Table 2).

**Table 2:** Comparisons of fatty acid composition (mg/g of subcutaneous fat) and carcass parameters among commercial types

	Commercial type				
	Salers	Pirenaica	Pirenaica	Holstein-Friesian	
	bulls (*n* = 13)	bulls (*n* = 37)	heifers (*n* = 29)	cows (*n* = 21)	*p*-value
Conformation	8.45 ± 0.37^b^	10.9 ± 0.3^a^	11.0 ± 0.3^a^	2.02 ± 0.64^c^	< 0.001
Fatness	5.79 ± 0.46^b^	4.52 ± 0.33^c^	7.47 ± 0.33^a^	1.96 ± 0.81^d^	< 0.001
14:0 ^s1^	31.6 ± 2.4^ab^	30.5 ± 1.7^b^	35.6 ± 1.8^a^	16.0 ± 4.2^c^	< 0.001
15:0 ^s2^	4.71 ± 0.33	4.07 ± 0.23	3.99 ± 0.24	3.13 ± 0.57	0.111
16:0 ^s3^	230 ± 11^ab^	217 ± 8^b^	246 ± 8^a^	170 ± 20^bc^	0.002
17:0 ^s4^	9.01 ± 0.71	7.48 ± 0.50	8.08 ± 0.51	5.54 ± 1.22	0.057
18:0 ^s5^	131 ± 11	119 ± 8	98.0 ± 8.4	129 ± 20	0.059
19:0 ^s6^	0.565 ± 0.074^a^	0.595 ± 0.052^a^	0.38 ± 0.05^b^	0.708 ± 0.129^a^	0.005
20:0 ^s7^	0.907 ± 0.119^ab^	0.816 ± 0.084^b^	0.468 ± 0.085^c^	1.34 ± 0.21^a^	< 0.001
9*c*-14:1 ^p1^	8.05 ± 1.27^b^	7.80 ± 0.90^b^	12.4 ± 0.9^a^	1.46 ± 2.21^c^	< 0.001
9*c*-15:1 ^p2^	0.208 ± 0.028	0.183 ± 0.020	0.206 ± 0.020	0.15 ± 0.05	0.572
9*c*-16:1 ^p3^	36.7 ± 3.7^b^	33.5 ± 2.6^b^	45.3 ± 2.7^a^	9.74 ± 6.51^c^	< 0.001
9*c*-17:1 ^p4^	6.73 ± 0.44^a^	5.24 ± 0.31^b^	6.96 ± 0.32^a^	2.15 ± 0.77^c^	< 0.001
9*c*-18:1 ^p5^	308 ± 14^a^	261 ± 10^b^	333 ± 10^a^	196 ± 24^c^	< 0.001
9*c*-19:1 ^p6^	0.987 ± 0.055^a^	0.789 ± 0.039^b^	0.801 ± 0.040^b^	0.795 ± 0.096^ab^	0.004
9*c*-20:1 ^p7^	0.726 ± 0.075	0.614 ± 0.053	0.728 ± 0.054	0.777 ± 0.130	0.231
6-8 *t*-18:1 ^s8^	3.14 ± 0.38^bc^	3.66 ± 0.27^ab^	4.12 ± 0.28^a^	1.83 ± 0.67^c^	0.010
11 *t*-18:1 ^s9^	10.3 ± 2.0	12.1 ± 1.4	8.20 ± 1.47	6.15 ± 3.55	0.162
12 *t*-18:1 ^s10^	2.26 ± 0.28	2.56 ± 0.20	2.71 ± 0.21	1.59 ± 0.50	0.174
13 *t*/14 *t*-18:1 ^s11^	4.37 ± 0.51^b^	5.23 ± 0.36^ab^	5.80 ± 0.37^a^	3.67 ± 0.89^ab^	0.029
15*c*-18:1 ^s12^	1.08 ± 0.16^c^	1.38 ± 0.11^b^	2.07 ± 0.11^a^	0.596 ± 0.271^c^	< 0.001
7 *t*,9*c*-18:2 ^p8^	0.813 ± 0.116^bc^	0.845 ± 0.082^b^	1.25 ± 0.08^a^	0.295 ± 0.201^c^	< 0.001
9*c*,11 *t*-18:2 ^p9^	3.11 ± 0.53	3.26 ± 0.376	3.53 ± 0.38	1.80 ± 0.92	0.453
9*c*,12 *t*-18:2 ^p10^	0.520 ± 0.067^b^	0.608 ± 0.048^b^	0.854 ± 0.048^a^	0.312 ± 0.118^b^	< 0.001
9*c*,13 *t*-18:2 ^p11^	0.963 ± 0.131^b^	1.07 ± 0.09^b^	1.61 ± 0.09^a^	0.567 ± 0.229^b^	< 0.001
9*c*,15*c*-18:2 ^p12^	0.461 ± 0.044^b^	0.341 ± 0.031^c^	0.587 ± 0.032^a^	0.266 ± 0.077^bc^	< 0.001
10 *t*,12*c*-18:2 ^i^	0.221 ± 0.041^b^	0.195 ± 0.029^b^	0.324 ± 0.030^a^	0.099 ± 0.072^b^	0.001
SFA	410 ± 21	382 ± 15	395 ± 15	328 ± 37	0.285
MUFA	427 ± 18^b^	379 ± 13^c^	489 ± 13^a^	245 ± 32^d^	< 0.001
*cis*-MUFA	385 ± 18^b^	331 ± 13^c^	430 ± 13^a^	221 ± 32^d^	< 0.001
*trans*-MUFA	42.2 ± 5.2^bc^	48.3 ± 3.6^b^	58.9 ± 3.7^a^	23.5 ± 9.0^c^	0.001
CLA	4.92 ± 0.56^bc^	5.22 ± 0.40^b^	6.42 ± 0.40^a^	2.68 ± 0.98^c^	0.002
PUFA	29.9 ± 2.1^a^	24.3 ± 1.5^bc^	24.4 ± 1.5^b^	14.9 ± 3.7^c^	0.009
n-6	27.7 ± 2.0^a^	22.2 ± 1.4^b^	22.1 ± 1.4^b^	12.8 ± 3.4^c^	0.005
n-3	2.09 ± 0.19	2.05 ± 0.14	2.15 ± 0.14	2.04 ± 0.33	0.942

Least square means ± standard deviations

*t trans*, *c cis*, *SFA* saturated fatty acids, *MUFA* monounsaturated fatty acids, *CLA* conjugated linoleic acid, *PUFA* polyunsaturated fatty acids

^s^ substrate; ^p^ product; ^1-12^Same superscript numbers indicate the substrate-product pairs

^i^ inhibitor of SCD enzyme [25]

^a,b,c,d^ Values within a row with different superscripts differ significantly at *P* < 0.05

SFA = 10:0 + 12:0 + 13:0 + 14:0 + 15:0 + 16:0 + 17:0 + 18:0 + 19:0 + 20:0 + 21:0 + 22:0 + 23:0 + 24:0

MUFA = 9*c*-14:1 + 9*c*-15:1 + 7*c*-16:1 + 9*c*-16:1 + 10*c*-16:1 + 11*c*-16:1 + 12*c*-16:1 + 13*c*-16:1 + 5*c*-17:1 + 7*c*-17:1 + 9*c*-17:1 + 9*c*-18:1 + 11*c*-18:1 + 12*c*-18:1 + 13*c*-18:1 + 14*c*-18:1 + 15*c*-18:1 + 16*c*-18:1 + 9*c*-19:1 + 11*c*-19:1 + 13*c*-19:1 + 9*c*-20:1 + 11*c*-20:1 + 6 *t*/7 *t*-16:1 + 8 *t*-16:1 + 9 *t*-16:1 + 10 *t*-16:1 + 11 *t*/12 *t*-16:1 + 4 *t*-18:1 + 5 *t*-18:1 + 6-8 *t*-18:1 + 9 *t*-18:1 + 10 *t*-18:1 + 11 *t*-18:1 + 12 *t*-18:1 + 13 *t*/14 *t*-18:1 + 15 *t*-18:1 + 16 *t*-18:1

CLA = 9*c*,11 *t*-18:2 + 7 *t*,9*c*-18:2 + 8*c*,10 *t*-18:2 + 9 *t*,11*c*-18:2 + 11*c*,13 *t*-18:2 + 10 *t*,12*c*-18:2 + 11 *t*,13*c*-18:2 + other *t*,*t*-18:2

PUFA = 18:2n-6 + 18:3n-6 + 20:2n-6 + 20:3n-6 + 20:4n-6 + 22:4n-6 + 18:3n-3 + 18:4n-3 + 20:5n-3 + 22:5n-3 + 22:6n-3 + 20:3n-9

### Gene expression

**Fig. 1:**
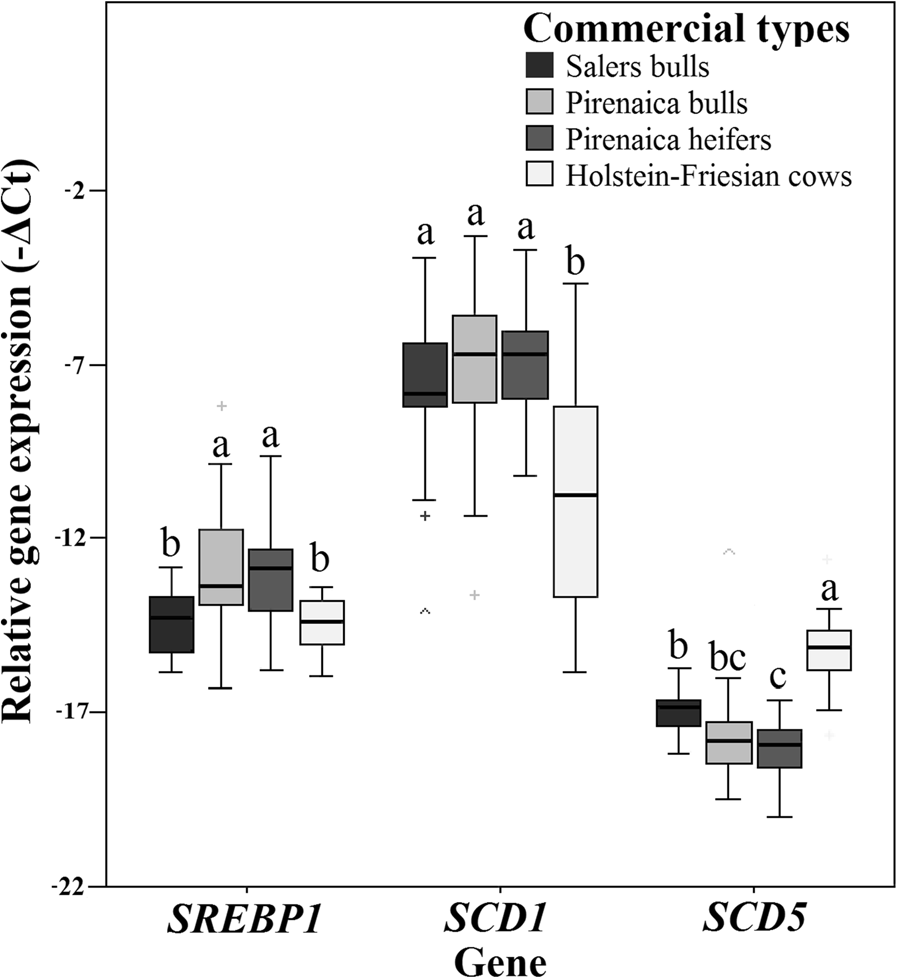
Box-plot showing the relative expression levels of *SCD1*, *SCD5* and *SREBP1* in subcutaneous fat samples from the cattle commercial types Salers, Pirenaica bulls, Pirenaica heifers and Holstein-Friesian heifers. The middle line in the box represents the median, upper and lower areas of the center box indicate the 75th and 25th percentiles respectively, and vertical bars indicate standard errors. Differences among commercial types are indicated by different letters (*P* < 0.05)

The relative mRNA expression levels of the lipogenic genes *SREBP1*, *SCD1*, and *SCD5* were similar in Pirenaica bulls and heifers (Fig. 1). Overall, *SCD1* expression was higher than *SREBP1* expression (*P* < 0.001) and *SCD5* expression (*P* < 0.001) in all commercial types, with average–ΔCt values of − 7.91, − 13.4, and − 17.2, respectively (Fig. 1). Differences among breeds were observed for each gene. The mRNA expression of *SCD1* was significantly higher in Salers (− 7.36) and Pirenaica cattle (average –ΔCt value of − 6.10) than Holstein-Friesian cows (− 13.8) (*P* < 0.001). In contrast, *SCD5* mRNA expression was lowest in Pirenaica bulls and heifers (average of − 17.8) among commercial types, highest in Holstein-Friesians cows (− 15.3), and at intermediate expression levels in Salers bulls (− 17.1; *P* < 0.001). In addition, expression of *SREBP1* mRNA was higher in Pirenaica bulls and heifers (average of − 12.73) than in the other commercial types (average − 14.8; *P* < 0.001).

### Relationships among gene expression and fatty acid composition data

**Fig. 2:**
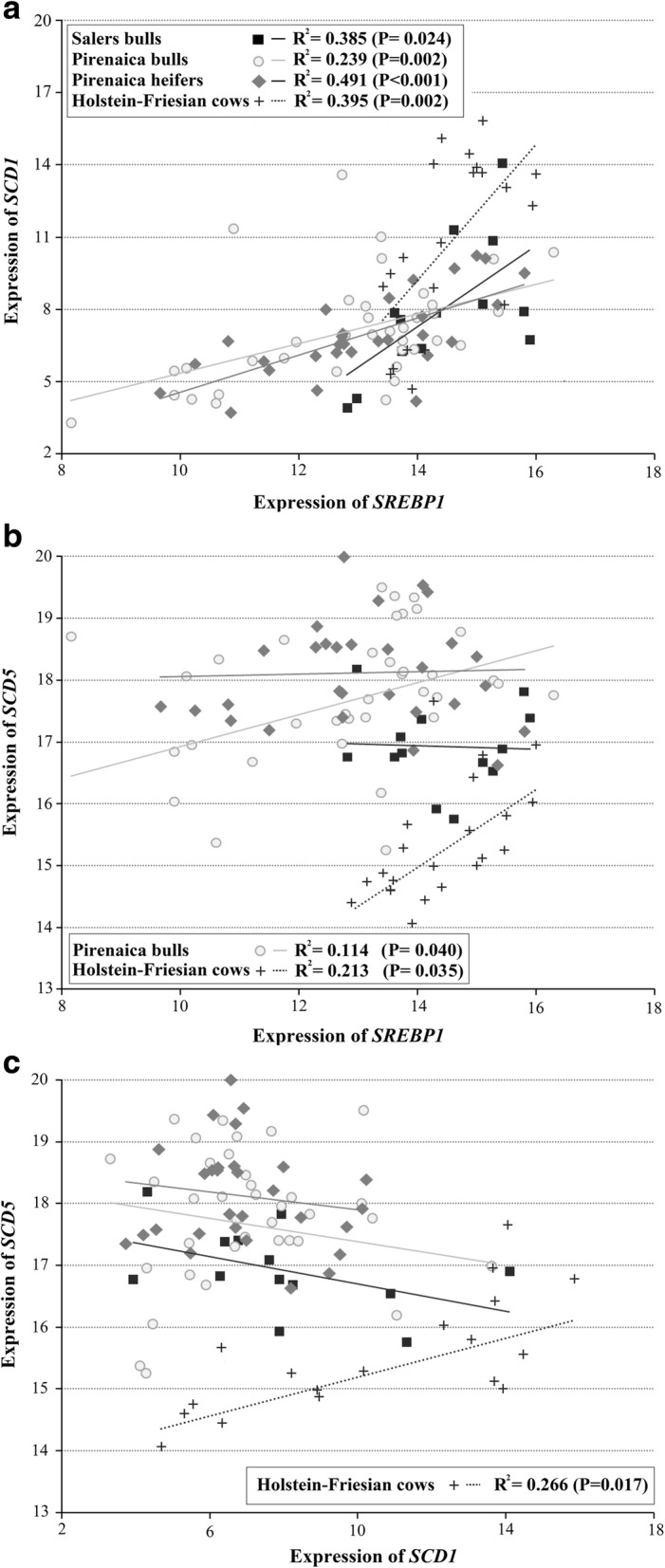
Estimated linear regression equations between (**a**) *SCD1* and *SREBP1*, (**b**) *SCD5* and *SREBP1*, and (**c**) *SCD5* and *SCD1*

Significant correlations were observed between studied gene pairs in all commercial types, with particularly strong correlation between *SCD1* and *SREBP1* (Fig. 2a). Pirenaica heifers showed the highest regression coefficient between *SCD1* and *SREBP1* among the commercial types (R^2^ = 0.491; *P* < 0.001). Salers bulls and Holstein-Friesian cows also showed relatively high regression coefficients between *SCD1* and *SREBP1* (R^2^ = 0.385; *P* = 0.024 and R^2^ = 0.395; *P* = 0.002, respectively), while Pirenaica bulls showed the lowest values (R^2^ = 0.239; *P* = 0.002). A positive correlation between *SCD5* and *SREBP1* (Fig. 2b) was observed in Pirenaica bulls (R^2^ = 0.114; *P* = 0.040) and Holstein-Friesian cows (R^2^ = 0.213; *P* = 0.035), while in Salers bulls and Pirenaica heifers was not (*P* > 0.05). No significant correlations were observed between *SCD5* and *SCD1* gene expression except for Holstein-Friesian cows (R^2^ = 0.266, *P* = 0.017; Fig. 2c).

**Fig. 3:**
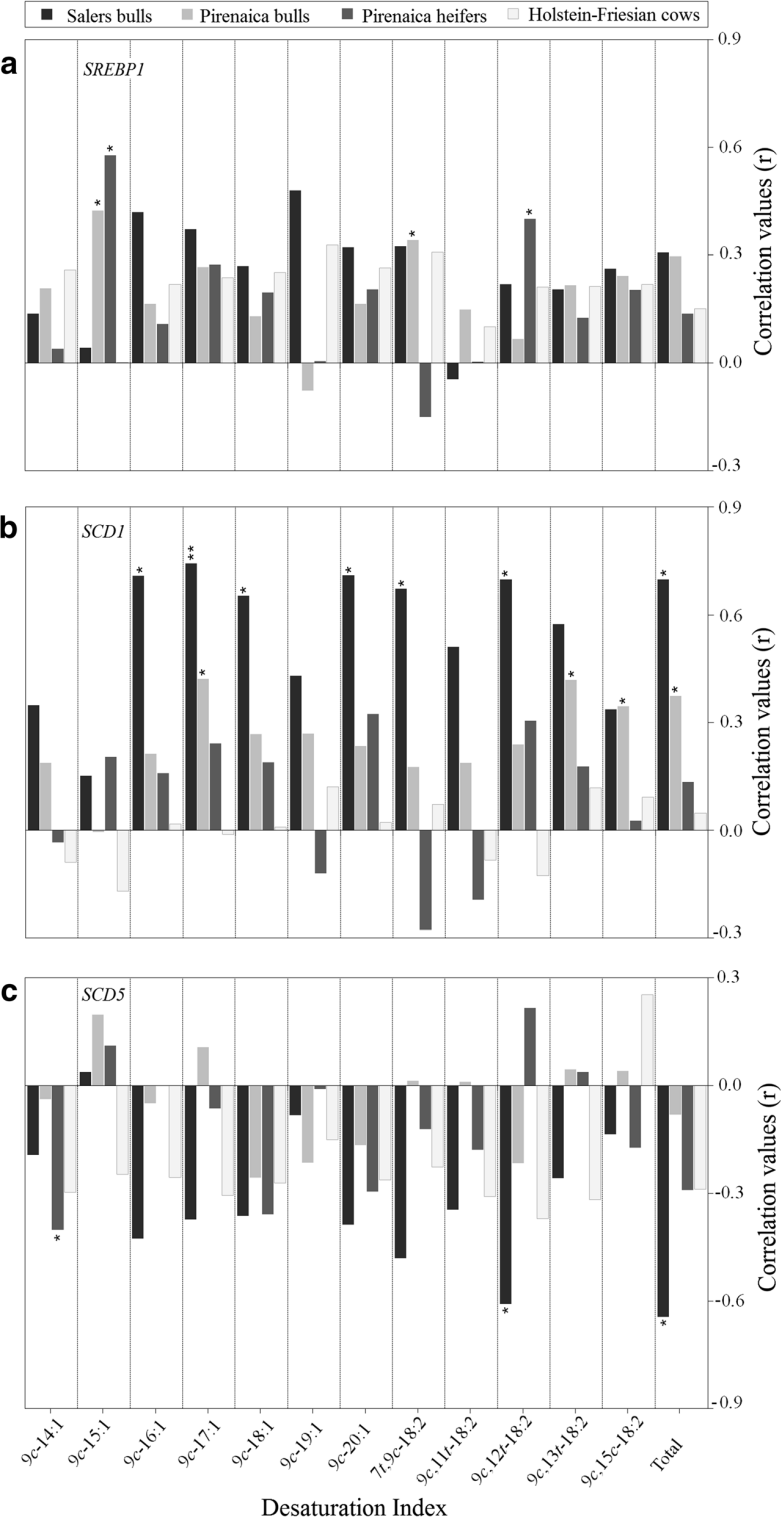
Partial correlations controlling for age and HCW between gene expression of *SREBP1* (**a**), *SCD1* (**b**), *SCD5* (**c**) and desaturation indexes calculated from fatty acid composition data of cattle commercial types. ^*^P < 0.05, ^**^P < 0.01. Total is sum of all individual DIs. Desaturation indexes were calculated as [SCD product]/([SCD substrate] + [SCD product])

In all commercial types, *SREBP*1 expression was positively correlated with the DI of most FA species, and correlations were significant for 9*c*-15:1 and 7 *t*,9*c*-18:2 in Pirenaica bulls and 9*c*-15:1 and 9*c*,12 *t*-18:2 in Pirenaica heifers (*P* < 0.05; Fig. 3a). In general, Salers bulls showed the highest positive correlations (*R* > 0.65) between *SCD*1 expression and DIs for 9*c*-16:1, 9*c*-17:1, 9*c*-18:1, 9*c*-20:1, 7 *t*,9*c*-18:2 and 9*c*,12 *t*-18:2 (Fig. 3b). Pirenaica bulls also showed significant positive correlations between *SCD*1 expression and DIs for 9*c*-17:1, 9*c*,13 *t*-18:2, and 9*c*,15*c*-18:2 DIs (*P* < 0.05), while Pirenaica heifers did not (*P* > 0.05). In contrast to *SREBP*1 and *SCD*1, there were few significant correlations between *SCD*5 and DIs among commercial types (Fig. 3c). A negative correlation was observed between *SCD5* and 9*c*,12 *t*-18:2 DI in Salers and 9*c*-14:1 DI in Pirenaica heifers (*P* < 0.05). Total DI was positively correlated with *SCD*1 in Salers (R > 0.65, *P* < 0.05) and Pirenaica bulls (*R* > 0.35, *P* < 0.05), but negatively correlated with *SCD*5 in Salers bulls (*R* > 0.60, *P* < 0.05).

## Discussion

Fat deposition and the FA composition of fat depots are controlled by a complex regulatory system including lipogenesis and lipolysis pathways. Adipose tissue is the main site for the storage of excess energy in the form of triacylglycerols, with the ∆9-desaturase product oleic acid (9*c*-18:1) being the predominant FA [29]. Therefore, ∆9-desaturase activity is critical for triglyceride storage in adipose tissue. While several pathways are involved in regulating FA composition, FAs produced from the precursors acetate and NADH, from the hydrolysis of triacylglycerols, and produced and deposited as rumen biohydrogenation metabolites can act as substrates for ∆9-desaturase. Adipose tissue develops in inter- and intra-muscular depots and both have a major impact on the quality and palatability of commercial beef. There is evidence for differential gene expression profiles in these two fat depots [30]. In this regard, the present study aimed to evaluate the regulation of *SCD* and *SREBP1*, genes strongly affecting the FA composition of subcutaneous adipose tissue, in three genetically diverse bovine breeds commercialized in the Basque region of northern Spain; Pirenaica, Salers and Holstein-Friesian [5, 31, 32]. Expression of *SCD1* did not differ significantly between Pirenaica bulls and heifers or among young cattle of Salers and Pirenaica. This may be partially explained by a similar feeding regimen, typically including concentrates, when meat production is the final purpose (Fig. 1). However, the content of Δ9 products, such as *cis*-MUFA, was higher in Salers bulls and Pirenaica heifers than corresponding bulls (Table 2). The Salers bulls and Pirenaica heifers, together with Holstein-Friesian cows, showed stronger correlations between *SCD1* and *SREBP*1 compared to Pirenaica bulls (Fig. 2). This suggests that, in Pirenaica breed, the FA composition is affected by the lipogenic gene regulation in a sex-dependent manner. Similarly, in a crossbred study, heifers exhibited higher *SCD1* mRNA levels and higher MUFA content than bulls in subcutaneous adipose tissue [33], and a possible effect of sex hormones on enzymatic systems affecting lipid metabolism has been suggested [34]. Indeed, the growth hormone, sexually differentiated in mammals, seems to increase *SREBP*1 and *SCD1* gene expression in females [35]. Alternatively, age and diet have been demonstrated to influence adipocyte development in Pirenaica bulls [36]. Hence, the activation of *SCD1* due to a potentially higher concentrate consumption [19, 37] agrees with the greater total MUFA content of Salers and Pirenaica, while higher MUFA content in Pirenaica heifers than bulls seems to be more sex-dependent (Table 2). The greater variability in *SCD1* expression within Holstein-Friesian cows compared to young Pirenaica and Salers (Fig. 1) could be related to the less homogeneous diet and older age of these animals. Nevertheless, the generally lower *SCD1* expression observed in Holstein-Friesian cows was also reported in other mature culled cows [4], in which linoleic acid (18:2n-6) was suggested as the primary agent depressing *SCD* gene expression in adipose tissue [38].

We detected variability in *SCD5* mRNA expression levels among breeds (*P* < 0.01) and generally greater expression of *SCD1* relative to *SCD5* in all breeds. Lengi and Corl (2007) [11] also reported over 40-fold greater expression of *SCD1* compared to *SCD5* in adipose tissue of bulls (albeit with unspecified feeding). Variation among breeds was observed in the expression of both *SCD* isoforms, especially between beef and dairy cattle breeds (Salers and Pirenaica vs. Holstein-Friesian), suggesting that even if FA differences are generally small, there may still be differences in the underlying lipogenic gene expression or enzyme profile [39].

These differences in *SCD1* and *SCD5* expression levels (Fig. 1) also suggest that *SCD5* expression is more breed dependent than *SCD1* expression. However, it is also possible that *SCD5* expression is more sensitive than *SCD1* expression to other environmental factors (i.e., feeding) that differ among commercial types. Our results also revealed a potential opposite association between *SCD* isoforms within each breed. In general, this opposite correlation of DIs with *SCD*5 and *SCD*1 expression levels suggests that regulatory factors that upregulate *SCD*1 also downregulate *SCD*5 (and vice versa). However, since both *SCD* isoforms are expressed in adipose tissue, both may contribute to the maintenance of desaturation. In contrast to Pirenaica and Salers, this opposite association between *SCD1* and *SCD5* was not observed in Holstein-Friesian cows, a breed selected intensively for dairy production and most often utilized for beef production as cull cows at no specific age. This opposite pattern was more evident when the Holstein-Friesian sample was stratified by age (data not shown). Furthermore, Holstein-Friesian cows may exhibit side effects of dairy selection that differentially affect the genetic sequences containing *SCD* genes, thereby influencing transcriptional regulation. Moreover, variability in the regulatory DNA sequences of *SCD* genes may confer differences in gene expression and physiological changes that could also explain the different correlation patterns with DIs observed among commercial types. 9*c*-14:1 DI has been reported as the best indicator of overall SCD enzyme activity by Corl et al. (2002) [40]. Feedstuffs are normally devoid of 9*c*-14:1 and, therefore, this FA is produced by de novo FA synthesis. In mammary gland, significant correlation between 9*c*-14:1 DI and *SCD1* expression was observed, whereas 9*c*-14:1 DI and *SCD5* correlation was not [41]. We did not observe significant correlation between 9*c*-14:1 DI and *SCD1,* similarly to a previous study in intestinal adipose tissue, skeletal muscle or mammary gland [42]. However, we observed that 9*c*-14:1 DI and *SCD5* were negatively correlated in subcutaneous adipose tissue of Pirenaica heifers (*P* < 0.05). Thus, these results suggest that *SCD* gene expression may directly affect 9*c*-14:1 content, but 9*c*-14:1 DI correlation with *SCD5* and *SCD1* might be breed and tissue specific as well.

As previously reported by Horton et al. [14], *SCD1* and *SREBP1* appear to be directly related as there was a significant linear association between these two genes in all commercial types studied (Fig. 2a). Differences in slope and coefficient of determination (R^2^) values, however, revealed variability in this relationship among commercial types. A previous study suggested that the FA synthesis pathway is regulated in a coordinated manner by the SREBP family of membrane-bound transcription factors, and regulation of *SCD1* by *SREBP1* via the SRE binding site of *SCD1* has been demonstrated [43].

Significant correlation between *SCD5* and *SREBP1* specifically observed in Pirenaica bulls and Holstein-Friesian cows suggest that *SCD5* expression may be more variable among commercial types than *SCD1* expression, possibly due to differences in regulation by *SREBP1*. For example, according to Lengi and Corl (2012) [16], the early growth response protein 2 (*EGR2*) and *SREBP1* may bind to the same DNA site of the bovine *SCD5* promoter. They observed that expression of *EGR2* or *SREBP1* did not increase endogenous *SCD5* mRNA expression but did activate a truncated bovine *SCD5* promoter luciferase reporter constructs in human JEG3 cells. Therefore, they attributed the lack of increase in *SCD5* expression to the presence of additional negative-regulation sites in this gene. In our case, the absence of significant differences in other commercial types could be due to breed or other environmental factors that could, in part, modulate these putative negative-regulation sites.

Among these commercial types of the Basque region, correlations between lipogenic genes (*SCD1* and *SREBP1*) and calculated DIs were stronger in Salers bulls than Pirenaica bulls and heifers (Fig. 3). In Holstein-Friesian cows, correlations were not significant (Fig. 3). However, *SREBP1* and *SCD1* correlations with DIs became significant (*P* < 0.05) when computed without age and HCW as covariates (data not shown). Moreover, correlations between *SCD1* expression and DIs were slightly higher in younger Holstein-Friesians, while *SCD5* correlations with DIs were higher in older Holstein-Friesians. This suggests an effect of age on gene expression−DI correlations in Holstein-Friesian cows. In addition to 16:1 and 18:1 [42], positive correlations between *SCD1* and calculated DIs were observed for other FA species, suggesting desaturase activity also targets minor FAs of subcutaneous fat. In this regard, DIs and MUFA content were more susceptible to the expression of lipogenic genes in Pirenaica heifers than bulls. Furthermore, the effect of lipogenic gene expression on DIs was stronger in Salers than Pirenaica heifers. Our findings are supported by a previous study [13] suggesting that the FA composition of subcutaneous adipose tissue is mainly dependent on genetic background, which may in turn indicate inter-breed differences in lipid metabolism. The effect of breed appears to be more strongly associated with *SREBP*1 expression level than *SCD1* or *SCD5* expression level (Fig. 1), whereas the underlying regulation of *SCD1* and *SCD5* could be responsible for inter-breed differences in DIs and FA profiles.

We also report an opposite effect of *SCD* isoforms on certain DI values, especially in Salers bulls. This stronger pattern may stem for a more homogeneous production system (Salers breeder, personal communication) that may reduce the influences of extraneous factors. Positive correlations between DIs and *SCD*1 (Fig. 2b) and contrasting negative correlations between DIs and *SCD*5 (Fig. 2c) are likely due to genetic compensation. Lower expression of one *SCD* isoform could well be compensated for by upregulation of the other isoform (Fig. 1). This compensation theory was previously suggested in *Caenorhabditis elegans* [44]. The reciprocal expression observed between different isoforms and the underlying epigenetic processes require further investigation.

The CLA isomer 10 *t*,12*c*-18:2 was examined because it was previously described as an important inhibitor of *SCD1* in dairy cattle [25]. In our study, although Pirenaica heifers accumulated the highest amounts of 10 *t*,12*c*-18:2 in subcutaneous adipose tissue (Table 2), no significant correlation was observed between 10 *t*,12*c*-18:2 and lipogenic gene expression (data not shown). Nevertheless, both isoforms may be differently regulated. In contrast to *SCD1*, which tends to be reduced by 10 *t*,12*c*-18:2 [45], *SCD5* appears to be more stable due to lack of an N-terminal PEST sequence for degradation [11]. However, further research is needed to establish relationships among DIs and SCD isoform mRNA expression levels, and to clarify the effects of 10 *t*,12*c*-18:2 on bovine adipose and muscle tissues. Analysis of lipogenic gene expression changes with dietary treatment in ruminant species as well as promoter sequencing would provide valuable insight into the regulation of these genes and their impact on the synthesis of MUFAs and PUFAs.

## Conclusion

The present study suggests that the differences in subcutaneous fat FA composition among bovine commercial types of the Basque region are related to genetic variability in lipogenic gene expression. The expression of lipogenic genes in Salers bulls showed clear effects on desaturation indexes and FA composition. All breeds show a strong correlation between *SREBP1* and *SCD1* expression. In addition, distinct correlations between *SCD* isoforms and DIs suggest a novel genetic compensation mechanism between *SCD1* and *SCD5* that warrants further investigation.

## Abbreviations

*c*: *Cis*
CLA: Conjugated linoleic acid
DI: Desaturation index
FA: Fatty acid
HCW: Hot carcass weight
MUFA: Monounsaturated fatty acids
PUFA: Polyunsaturated fatty acids
SCD: Stearoyl-CoA desaturase
SFA: Saturated fatty acids
SREBP: Sterol regulatory element-binding protein
*t*: *Trans*
UFA: Unsaturated fatty acids

## References

[1] World Health Organization (2003). Diet, nutrition and the prevention of chronic diseases: report of a joint WHO/FAO expert consultation.

[2] Burlingame B, Nishida C, Uauy R, Weisell R (2009). Fats and fatty acids in human nutrition. Joint FAO/WHO expert consultation. Ann Nutr Metab.

[3] Wood JD, Richardson RI, Nute GR, Fisher AV, Campo MM, Kasapidou E (2004). Effects of fatty acids on meat quality: a review. Meat Sci.

[4] Brooks MA, Choi CW, Lunt DK, Kawachi H, Smith SB (2011). Subcutaneous and intramuscular adipose tissue stearoyl-coenzyme a desaturase gene expression and fatty acid composition in calf- and yearling-fed Angus steers. J Anim Sci.

[5] Gamarra D, Lopez-Oceja A, de Pancorbo M (2017). Genetic characterization and founder effect analysis of recently introduced Salers cattle breed population. Animal.

[6] St John LC, Lunt DK, Smith SB (1991). Fatty acid elongation and desaturation enzyme activities of bovine liver and subcutaneous adipose tissue microsomes. J Anim Sci.

[7] Taniguchi M, Mannen H, Oyama K, Shimakura Y, Oka A, Watanabe H, Kojima T, Komatsu M, Harper GS, Tsuji S (2004). Differences in stearoyl-CoA desaturase mRNA levels between Japanese black and Holstein cattle. Livest Prod Sci.

[8] Kgwatalala PM, Ibeagha-Awemu EM, Hayes JF, Zhao X (2007). Single nucleotide polymorphisms in the open reading frame of the stearoyl-CoA desaturase gene and resulting genetic variants in Canadian Holstein and Jersey cows. DNA Seq.

[9] Barton L, Kott T, Bures D, Rehak D, Zahradkova R, Kottova B (2010). The polymorphisms of stearoyl-CoA desaturase (SCD1) and sterol regulatory element binding protein-1 (SREBP-1) genes and their association with the fatty acid profile of muscle and subcutaneous fat in Fleckvieh bulls. Meat Sci.

[10] Li C, Aldai N, Vinsky M, Dugan ME, McAllister TA (2012). Association analyses of single nucleotide polymorphisms in bovine stearoyl-CoA desaturase and fatty acid synthase genes with fatty acid composition in commercial cross-bred beef steers. Anim Genet.

[11] Lengi AJ, Corl BA (2007). Identification and characterization of a novel bovine stearoyl-CoA desaturase isoform with homology to human SCD5. Lipids.

[12] Rincon G, Islas-Trejo A, Castillo AR, Bauman DE, German BJ, Medrano JF (2012). Polymorphisms in genes in the SREBP1 signalling pathway and SCD are associated with milk fatty acid composition in Holstein cattle. J Dairy Res..

[13] da Costa AS, Pires VM, Fontes CM, Prates JA (2013). Expression of genes controlling fat deposition in two genetically diverse beef cattle breeds fed high or low silage diets. BMC Vet Res.

[14] Horton JD, Goldstein JL, Brown MS (2002). SREBPs: activators of the complete program of cholesterol and fatty acid synthesis in the liver. J Clin Invest.

[15] Hoashi S, Ashida N, Ohsaki H, Utsugi T, Sasazaki S, Taniguchi M, Oyama K, Mukai F, Mannen H (2007). Genotype of bovine sterol regulatory element binding protein-1 (SREBP-1) is associated with fatty acid composition in Japanese black cattle. Mamm Genome.

[16] Lengi AJ, Corl BA (2012). Regulation of the bovine SCD5 promoter by EGR2 and SREBP1. Biochem Biophys Res Commun.

[17] Widmann P, Nuernberg K, Kuehn C, Weikard R (2011). Association of an ACSL1 gene variant with polyunsaturated fatty acids in bovine skeletal muscle. BMC Genet.

[18] Jones OR, Wang J (2010). COLONY: a program for parentage and sibship inference from multilocus genotype data. Mol Ecol Resour.

[19] Aurtenetxe M, Belaunzaran X, Bravo-Lamas L, Gamarra D, Barron LJR, Aldai N (2017). Caracterización comercial y nutricional de la grasa subcutánea de terneros y vacas de desvieje sacrificados en la Comunidad Autónoma del País Vasco. Archivos de Zootecnia.

[20] The Commission of the European Communities. Commission regulation (EEC) no 103/2006 of 20 January 2006 adopting additional provisions for the application of the Community scale for the classification of carcases of adult bovine animals. Off J Eur Communities. 2006;49:6–8.

[21] Kramer JK, Fellner V, Dugan ME, Sauer FD, Mossoba MM, Yurawecz MP (1997). Evaluating acid and base catalysts in the methylation of milk and rumen fatty acids with special emphasis on conjugated dienes and total trans fatty acids. Lipids.

[22] Kramer JK, Hernandez M, Cruz-Hernandez C, Kraft J, Dugan ME (2008). Combining results of two GC separations partly achieves except CLA isomers of milk fat as demonstrated using ag-ion SPE fractionation. Lipids.

[23] Delmonte P, Fardin-Kia AR, Kramer JKG, Mossoba MM, Sidisky L, Tyburczy C, Rader JI (2012). Evaluation of highly polar ionic liquid gas chromatographic column for the determination of the fatty acids in milk fat. J Chromatogr A.

[24] Bravo-Lamas L, Barron LJ, Kramer JK, Etaio I, Aldai N (2016). Characterization of the fatty acid composition of lamb commercially available in northern Spain: emphasis on the trans-18:1 and CLA content and profile. Meat Sci.

[25] Baumgard LH, Matitashvili E, Corl BA, Dwyer DA, Bauman DE (2002). Trans-10, Cis-12 conjugated linoleic acid decreases Lipogenic rates and expression of genes involved in milk lipid synthesis in dairy cows. J Dairy Sci.

[26] Destaillats F, Trottier JP, Galvez JMG, Angers P (2005). Analysis of alpha-linolenic acid biohydrogenation intermediates in milk fat with emphasis on conjugated linolenic acids. J Dairy Sci.

[27] Zhu LJ, Altmann SW (2005). mRNA and 18S-RNA coapplication-reverse transcription for quantitative gene expression analysis. Anal Biochem.

[28] Ramakers C, Ruijter JM, Lekanne Deprez RH, Moorman AFM (2003). Assumption-free analysis of quantitative real-time polymerase chain reaction (PCR) data. Neurosci Lett.

[29] Mauvoisin D, Mounier C (2011). Hormonal and nutritional regulation of SCD1 gene expression. Biochimie.

[30] Bong JJ, Cho KK, Baik M (2010). Comparison of gene expression profiling between bovine subcutaneous and intramuscular adipose tissues by serial analysis of gene expression. Cell Biol Int.

[31] Martín-Burriel I, Rodellar C, Cañon J, Cortés O, Dunner S, Landi V, Martínez-Martínez A, Gama LT, Ginja C, Penedo MC, Sanz A, Zaragoza P, Delgado JV (2011). Genetic diversity, structure, and breed relationships in Iberian cattle. J Anim Sci.

[32] Lopez-Oceja A, Muro-Verde A, Gamarra D, Cardoso S, de Pancorbo MM. New Q lineage found in bovine (Bos taurus) of Iberian peninsula. Mitochondrial DNA. 2016;27:3597–601. 10.3109/19401736.2015.1079823 26554433

[33] Barton L, Bureš D, Kott T, Rehák D (2011). Effect of sex and age on bovine muscle and adipose fatty acid composition and stearoyl-CoA desaturase mRNA expression. Meat Sci.

[34] Zhang Y-Y, Zan L-S, Wang H-B, Xin Y-P, Adoligbe CM, Ujan JA (2010). Effect of sex on meat quality characteristics of Qinchuan cattle. African J Biotechnol.

[35] Ameen C, Linden D, Larsson BM, Mode A, Holmang A, Oscarsson J (2004). Effects of gender and GH secretory pattern on sterol regulatory element-binding protein-1c and its target genes in rat liver. Am J Physiol Endocrinol Metab.

[36] Soret B, Mendizabal A, Arana A, Alfonso L (2016). Expression of genes involved in adipogenesis and lipid metabolism in subcutaneous adipose tissue and longissimus muscle in low-marbled Pirenaica beef cattle. Animal.

[37] Smith S, Gill C, Lunt D, Brooks M (2009). Regulation of fat and fatty acid composition in beef cattle. Asian-Australasian. J Anim Sci.

[38] Smith SB, Ntambi J (2013). Functional development of Stearoyl-CoA desaturase gene expression in livestock species. Stearoyl-CoA desaturase genes lipid Metab.

[39] De Smet S, Raes K, Demeyer D (2004). Meat fatty acid composition as affected by fatness and genetic factors: a review. Anim Res.

[40] Corl BA, Baumgard LH, Griinari JM, Delmonte P, Morehouse KM, Yurawecz MP, Bauman DE (2002). Trans-7, cis-9 CLA is synthesized endogenously by ∆9-desaturase in dairy cows. Lipids.

[41] Jacobs AA, van Baal J, Smits MA, Taweel HZ, Hendriks WH, van Vuuren AM, Dijkstra J (2011). Effects of feeding rapeseed oil, soybean oil, or linseed oil on stearoyl-CoA desaturase expression in the mammary gland of dairy cows. J Dairy Sci.

[42] Rezamand P, Watts JS, Yavah KM, Mosley EE, Ma L, Corl BA, McGuire MA (2014). Relationship between stearoyl-CoA desaturase 1 gene expression, relative protein abundance, and its fatty acid products in bovine tissues. J Dairy Res.

[43] Tabor DE, Kim JB, Spiegelman BM, Edwards PA (1999). Identification of conserved cis elements and transcription factors required for sterol regulated transcription of stearoyl CoA desaturase 1 and 2. J Biol Chem.

[44] Brock TJ, Browse J, Watts JL (2006). Genetic regulation of unsaturated fatty acid composition in C. Elegans. PLoS Genet.

[45] Gervais R, McFadden JW, Lengi AJ, Corl BA, Chouinard PY (2009). Effects of intravenous infusion of trans-10, cis-12 18:2 on mammary lipid metabolism in lactating dairy cows. J Dairy Sci.

